# Comparative analysis of biological aspects of *Leishmania infantum* strains

**DOI:** 10.1371/journal.pone.0230545

**Published:** 2020-12-03

**Authors:** Taiana Ferreira-Paes, Karen dos Santos Charret, Merienny Ramos da Silva Ribeiro, Raquel Ferreira Rodrigues, Leonor Laura Leon

**Affiliations:** 1 Laboratório de Bioquímica de Tripanosomatídeos, Instituto Oswaldo Cruz/Fundação Oswaldo Cruz, Rio de Janeiro, Brazil; 2 Laboratório de Biologia Molecular e Doenças Endêmicas, Instituto Oswaldo Cruz/Fundação Oswaldo Cruz, Rio de Janeiro, Brazil; Instituto Oswaldo Cruz, BRAZIL

## Abstract

*Leishmania infantum infantum* (LII) is one of the species that causes visceral leishmaniasis (VL) in the Old World, while *L*. *infantum chagasi* (LIC) is present in the New World. Few studies address biological differences or the behavior of these strains during infection. These parasites live inside cells of their hosts, continuously evading microbicidal mechanisms and modulating the immune responses of these cells. One of the mechanisms used by these protozoa involves the L-arginine metabolism. Understanding the differences between *Leishmania* species and establishing an improved murine model for study of leishmaniasis are matters of extreme importance. Thereby, the objectives of this work were to analyze the biological and molecular differences between two *Leishmania infantum* strains (LII and LIC) and the degree of susceptibility to infection of mice with different genetic backgrounds. The infectivity *in vivo* and *in vitro* of LII and LIC strains was evaluated in BALB/c and Swiss Webster mice, as well the NOS and ARG activities. The LII strain was more infective than the LIC strain both *in vivo* and *in vitro*. In animals infected by the LII and LIC strains, differences in NOS and ARG activities occurred. *In vitro*, promastigotes of LII isolated from BALB/c and Swiss Webster mice showed higher ARG activity than LIC promastigotes during the growth curve. However, no difference was observed in intracellular NO production by promastigotes of these strains. The ARG gene sequences were compared, and those of both strains were identical. However, despite the similarity, the strains showed different expression levels of this gene. It can be concluded that although *L*. *chagasi* strains are considered identical to *L*. *infantum* strains from a molecular point of view, these strains have different biological behavior.

## Introduction

Leishmaniases are a group of infectious diseases found worldwide and are caused by protozoa of the genus *Leishmania*. These diseases are distributed in 200 countries or territories, and approximately 20,000 deaths each year are attributed to them. These diseases can manifest in various forms with different symptoms depending on the infecting species and the host’s immune response. Visceral leishmaniasis (VL) is the most lethal manifestation, with almost 0.5 million new cases each year. If not treated, the mortality rate can reach 100% in two years [1, 2].

*Leishmania infantum* is one of the species that causes VL and is present in the Mediterranean Basin, the Middle East and South Asia. Some years ago, *L*. *chagasi* was considered a new species of *Leishmania* causing visceral leishmaniasis in the New World. However, after genetic sequencing studies, *L*. *chagasi* was determined to be identical to *L*. *infantum*, being denominated *L*. *infantum* (syn. *L*. *chagasi*) [3]. Nevertheless, other studies have considered these strains as two subspecies, *L*. *infantum infantum* in the Old World and *L*. *infantum chagasi* in the New World, based on antigenic differences [4]. For leishmaniases studies, knowing the particularities of different species of *Leishmania* is extremely important. Few studies have demonstrated biological differences between parasites *of L*. *infantum infantum* and *L*. *infantum chagasi* or differences in their interactions with host cells. Thus, comparative studies between the two parasites are important and widely needed.

Our previous studies have reported a possible relationship between infectivity and the metabolic pathway of L-arginine not only in *L*. *infantum chagasi* but also in *L*. *amazonensis* and *L*. *braziliensis* [5–7]. L-arginine is metabolized mainly by nitric oxide synthase (NOS) and arginase (ARG) [8], and the amount of this accessible amino acid is critical for *Leishmania* proliferation or death [9, 10]. NOS and ARG can act directly on the intracellular fate of the parasite in macrophages; NOS activity results in nitric oxide (NO) production, which is harmful to the parasite, whereas ARG produces L-ornithine, which is essential for parasite proliferation [11–14]. As reported, L-arginine metabolism is important during *Leishmania* infection. However, how it functions in the host-parasite interaction and in strains from other regions is still unclear.

In experimental chemotherapy studies, the choice of the animal model is a primary issue. Hamsters are one of the models used in VL studies. However, the use of these animals is still limited due to high maintenance costs and manipulation difficulties. In addition, there is a difficulty in finding specific reagents, such as cellular markers for immunological assays [15, 16]. Thus, mice are generally the chosen models due to the ease of manipulation and the similarity to the human genome [17]. This similarity allows the use of these animals to mimic human leishmaniasis manifestations [18]. Resistance/susceptibility to *Leishmania major* infection has been associated with the balance of Th1 and Th2 immune responses [19–21]. However, for other *Leishmania* species, the profile is not so clear-cut, hampering the study of the pathophysiology of the disease and the development of novel drugs. BALB/c mice (inbred) are highly susceptible to infection by *Leishmania* spp. since they present a Th2 immune response and develop chronic disease a few weeks post-infection. In addition, due to the genetic variation of the human population, infections are quite heterogeneous, which makes it difficult to trace similarities with infections in inbred mice. In accordance with the above information, the aim of this work was to demonstrate biological differences between these two *L*. *infantum* strains *in vivo* and *in vitro*, including differences in L-arginine metabolism, which are very important for parasite survival. Moreover, this study compared the degree of susceptibility of mice with different genetic backgrounds to infection by these parasites.

In this work, it was demonstrated that *L*. *infantum infantum* and *L*. *infantum chagasi* present different behaviors during *in vivo* and *in vitro* infections. In addition, BALB/c and Swiss Webster mice have different susceptibilities to infection by these strains.

## Materials and methods

### Parasites

Two parasite strains were used: *Leishmania infantum infantum* (MHOM/MA/67/ITMAP263); hereafter called LII) and *Leishmania infantum chagasi* (MCAN/BR/97/P142); hereafter called LIC). In all protocols, infective isolates newly isolated from infected BALB/c and Swiss Webster mice were used. Promastigote forms were cultured at 26°C in Schneider’s Insect Medium (Sigma Aldrich, St. Louis, MO, USA) supplemented with 20% fetal calf serum (FCS), 100 U/mL Penicillin G potassium and 100 μg/mL streptomycin, at pH 6.9.

### Mice infection

Female BALB/c and Swiss Webster mice (6–8 weeks of age) were obtained from Instituto de Ciência e Tecnologia em Biomodelos (ICTB, FIOCRUZ, Rio de Janeiro, Brazil). All procedures involving animals were approved by the Ethics Committee on the Use of Animals at FIOCRUZ (CEUA LW-026/15). Six groups of animals (5 mice/group) were infected or not by an intraperitoneal injection of 1 x 10^8^/100 μL stationary-phase *Leishmania infantum* promastigotes as follows: Group 1: noninfected BALB/c mice (Control); Group 2: BALB/c mice infected by the LIC strain; Group 3: BALB/c mice infected by the LII strain; Group 4: noninfected Swiss Webster mice (Control); Group 5: Swiss Webster mice infected by the LIC strain; and Group 6: Swiss Webster mice infected by the LII strain. The animals were maintained for 30 or 60 days post-infection (dpi), when they were euthanized in a CO_2_ chamber and the organs (spleen and liver) were aseptically removed for further analysis.

### Limiting Dilution Analysis (LDA)

Parasite quantification in the spleen and liver was performed by the limiting dilution method. Mice were euthanized at 30 or 60 dpi, and the organs were aseptically removed, weighed and homogenized in Schneider’s medium supplemented with 20% FCS. The cells were placed in a 96-well plate containing Schneider’s medium plus 20% FCS and serially diluted. For 7 days, the plates were maintained at 26°C, and the wells were examined daily using an inverted microscope. The number of parasites/mg of tissue was estimated based on the total weight of the removed tissue and the parasite load in the serial dilution according to Taswell [22, 23].

### NOS activity in spleen and liver cultures

Nitric oxide (NO) present in the supernatants of cultures was evaluated indirectly by Green’s method [24]. Briefly, cells from infected organs were maintained in culture for 48 h. Afterward, 100 μL of supernatant were collected and mixed (v/v) with Griess reagent (0.1% N-1-naphthyl-ethylenediamine dihydrochloride in a solution of 5% phosphoric acid and 1% sulfanilamide). After 10 min at room temperature, the NO production was measured at 540 nm, using sodium nitrite in Schneider’s Insect Medium in a concentration range of 0.78 to 200 μM as the standard.

### ARG activity in spleen and liver cultures

Infected organ cells (1 x 10^7^) were treated as previously described by Corraliza and collaborators (1994) [25]. Briefly, spleen and liver cells were previously washed in sucrose and KCL solution, and an anti-proteolytic buffer was added. After cell lysis, L-arginine (0.5 M) at pH 9.7 was added. The samples were incubated at 37°C, and the reaction was stopped by the addition of an acidic solution (H_2_SO_4_, H_3_PO_4_ and water [1:3:7]). The amount of urea produced was measured by adding 25 μL 9% α-isonitrosopropiophenone in ethanol and heating at 100°C for 45 min. After 10 min in the dark, the absorbance was determined at 540 nm, using a urea solution in Schneider’s Insect Medium in a concentration range of 1.5 to 30 μg/mL as the standard.

### Promastigote proliferation

Promastigotes were obtained from amastigotes freshly isolated from the four infected experimental groups, named LIC.B (*L*. *infantum chagasi* isolated from BALB/c mice), LII.B (*L*. *infantum infantum* isolated from BALB/c mice), LIC.S *(L*. *infantum chagasi* isolated from Swiss Webster), and LII.S *(L*. *infantum infantum* isolated from Swiss Webster). Promastigotes with up to 5 passages were cultured as previously described. The initial inoculum used was 5 x 10^5^ promastigotes/mL, and the cultures were counted daily for up to 7 days. Cells and supernatant were collected for use in the determination of the percent of metacyclic forms, the infectivity to macrophages, the activity of NOS and ARG gene sequencing and expression.

### Metacyclogenesis of promastigotes

To compare the percentage of metacyclic parasites between the LIC.B, LII.B, LIC.S and LII.S groups during *in vitro* proliferation assay, a complement lysis test was performed. At 48, 72 and 96 h of the growth curve, the parasites were collected and washed in PBS, and the suspension was adjusted to 1 x 10^6^ parasites/mL in PBS and was incubated with 20% human complement (Sigma Aldrich). After 30 min, the number of resistant parasites was counted, and the percentage of metacyclic cells was calculated.

### Infectivity to peritoneal macrophages

Peritoneal macrophages were removed from BALB/c and Swiss Webster mice to evaluate the infectivity of the four isolates of the two *Leishmania* strains (LIC.B, LII.S, LIC.B and LIC.S). Mouse peritoneal cavities were washed with ice-cold RPMI 1640 medium (Sigma Aldrich) supplemented with 10% FCS and 2 mM L-glutamine. The cells were adjusted to 2 x 10^5^ macrophages/well (0.4 mL/well), placed in Lab-Tek eight-chamber slides and maintained for 1 h at 37°C with 5% CO_2_. Stationary-phase promastigotes were added to the cell cultures at a ratio of 5:1 (parasites/macrophage) and maintained overnight, followed by the removal of noninternalized parasites with successive washes with RPMI 1640 medium. Cultures were kept for 24 or 72 h, and cells were stained with Fast Panotic (Laborclin, Pinhais, PR, Brasil). Using light microscopy, the percentage of infected macrophages was determined. On each coverslip, at least 200 cells were randomly counted in triplicate. The infection index was calculated by the following formula:

$$\text{Infection}\;\text{index}=\frac{\left(\%\;\text{of}\;\text{infected}\;\text{macrophages}\;\text{x}\;\text{number}\;\text{of}\;\text{amastigotes}\right)}{\text{total}\;\text{number}\;\text{of}\;\text{macrophages}}$$

### NOS and ARG activities in promastigotes

To evaluate the NO production within promastigotes (LIC.B, LII.S, LIC.B and LIC.S), the parasites (2 x 10^6^/mL) were incubated with 200 μL of L-arginine solution and 5 μM 4,5-diaminofluorescein diacetate (DAF-2DA–Sigma) for 2 h at room temperature. The resulting fluorescent compound was measured by fluorimetry with an emission wavelength at 485 nm and an excitation wavelength at 530 nm [26, 27]. The assay specificity was confirmed using RAW 264.7 cells as a positive control. To analyze the ARG activity, promastigotes (1 x 10^7^) cultivated in Schneider’s medium plus 20% FCS were harvested at 48, 72 and 96 h. The amount of urea produced by them was measured as previously described.

### RNA purification, cDNA synthesis and qPCR of ARG

Total RNA was extracted from 10^7^ promastigotes of the four isolates, LIC.B, LII.S, LIC.B and LIC.S, using TRIzol reagent (Invitrogen, Carlsbad, CA, EUA) following the manufacturer's protocol. RNA (2 μg) was reverse transcribed through the GoScript™ Reverse Transcription System performed with Oligo(dT)_15_ (Promega, Madison, Wisconsin, EUA) according to the manufacturer’s instructions. The resultant cDNA was quantified in a Qubit 2.0 Fluorimeter (Thermo Fischer) and kept at - 20°C. Gene expression by qPCR was evaluated using a ViiA™ 7 Real-Time PCR System (Applied Biosystems, Carlsbad, CA, USA). All reactions were performed as biological triplicates and technical duplicates using a GoTaq® qPCR Master Mix Kit (Promega). The primers were designed according to the ARG gene sequence (LinJ.35.1490) obtained from the TriTrypDB.org platform (ARG: PF– 5’ GTGTGGTACGGTCTCCGGTA 3'; PR– 5’ GTGTGGTACGGTCTCCGGTA 3’). Alpha-tubulin and GAPDH were used as housekeeper genes (Alpha-tubulin: PF– 5’ CAGGTGGTGTCGTCTCTGAC 3’; PR– 5’ TAGCTCGTCAGCACGAAGTG 3’). Fluorescence readings were performed under the following conditions: pre-incubation at 95°C for 10 min, 40 cycles of amplification at 95°C for 30 s, and 60°C for 60 s. Gene expression analyses were performed using QuantStudio™ Software V1.2 (Applied Biosystems) based on ΔCt methods.

### Sequencing of the ARG gene

The ARG gene was amplified by conventional PCR from promastigote DNA. DNA from LIC.B, LII.S, LIC.B and LIC.S parasites was extracted using the Genomic DNA Purification Kit protocol (Thermo Fisher). Conventional PCR was performed with 50 ng DNA, GoTaq® Hot Start Master Mix 2x (Promega), specific primers (5’ CGCATATGATGGAGCACGTGCA 3’ and 5’ CGGGATCCCTACAGTTTGGCG 3’) and DEPC-treated water up to 25 μL. PCR amplification was performed with a programmable thermal cycler (Applied Biosystems). The amplification protocol was carried out as follows: 1 cycle at 95°C; 30 cycles of 30 s at 94°C, 30 s at 58°C, and 40 s at 72°C; and 1 cycle of 5 min at 72°C. Next, 200 ng of purified DNA and specific primers (described above) were used for Sanger sequencing using a Sequence Scanner (Applied Biosystems). The results were analyzed using the BioEdit program (Ibis Biosciences, Carlsbad, CA, USA).

### Statistical analysis

The means and standard deviations were determined from at least three independent experiments. Statistical analyses were performed with the program GraphPad Prism 5 (GraphPad Software, USA). The ANOVA test was applied followed by Tukey’s post-test, and p values less than 0.05 were considered significant.

## Results

### Parasite load

**Fig 1:**
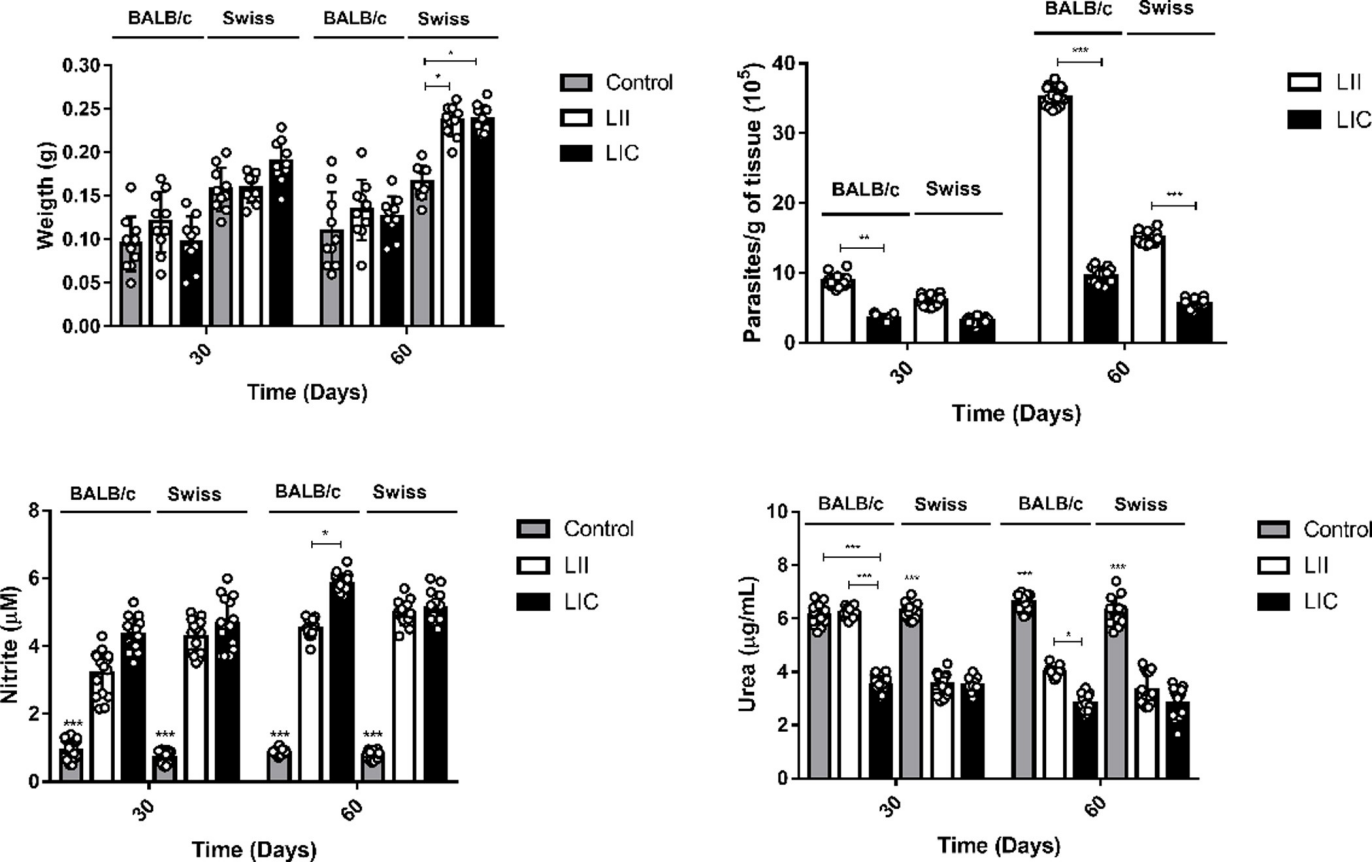
Evaluation of *in vivo* infection of two *L*. *infantum* strains. (A) Spleen weights of BALB/c and Swiss Webster mice infected by *L*. *infantum strains*. (B) Parasite load in the spleens of infected mice. (C) Nitrite levels (NOS activity) of spleen cell cultures. (D) Urea levels (ARG activity) of spleen cell cultures. BALB/c and Swiss Webster mouse infections were maintained for 30 or 60 dpi. The number of parasites/mg of tissue was estimated based on the total weight of the spleen removed and the parasite load in the serial dilution. The nitrite and urea levels were measured by spectrophotometry at 540 nm. Control: noninfected mice; LII (*L*. *infantum infantum*); LIC (*L*. *infantum chagasi*). *p ≤ 0.05; **p ≤ 0.0009; ***p < 0.0001. The values are represented by mean ± standard deviation of 3 independent experiments with 5 animals in each group.

Initially, BALB/c and Swiss Webster mice were infected (i.p.) with 1 x 10^8^/100 μL promastigotes of *L*. *infantum infantum* (LII) and *L*. *infantum chagasi* (LIC). Mice were kept for 30 or 60 days post-infection (dpi) until euthanasia. Total spleen weight was analyzed, and no alterations were observed in BALB/c mice infected with the LIC or LII strain at 30 and 60 dpi. In Swiss Webster mice infected with LII and LIC strains at 60 dpi, the spleen weight was significantly (p ≤ 0.05) higher than in noninfected counterparts (Fig 1A). No significant difference was observed in the liver weight between noninfected and corresponding infected groups. The LDAs of the spleen and liver cells of infected mice showed significant differences in the parasitic load among the different infected groups. In the spleen, this parameter was significantly higher at 60 dpi than at 30 dpi in three experimental groups, except in Swiss Webster mice infected by the LIC strain (Fig 1B). Comparing the LII and LIC strains, the former was always more infective in both mouse lineages. In BALB/c mice at 60 dpi, the LII strain was approximately 3 times more infective than the LIC strain. In the liver, although there were lower amounts of parasite than in the spleen, a greater parasite burden was also detected in the case of LII infection (S1A Fig).

### *In vivo* NOS and ARG activities

Since NOS and ARG enzymes are regulated by cytokines related to Th1- and Th2-type immunological response profiles, their activities may indicate the susceptibility/resistance to infection in different murine models [28]. After 48 h of incubation of the spleen and liver cell cultures, supernatants were used to indirectly quantify the activities of NOS (by nitrite levels) and ARG (by urea production). As expected, there were significant increases (p < 0.0001) in nitrite levels due to *Leishmania* infection at both 30 and 60 dpi. In BALB/c mice, higher NO levels in spleen cultures were observed after infection with the LIC strain when compared to the LII strain, with the differences for the two parasite strains being statistically significant (p ≤ 0.05) at 60 dpi (Fig 1C). In Swiss Webster mice, there were no significant differences between the infections with LIC and LII strains. In liver cultures from BALB/c mice, infection by the LIC strain showed higher nitrite levels than infection by the LII strain at both 30 and 60 dpi. In Swiss Webster mice, significant differences (p ≤ 0.009) were observed only at 60 dpi, when LIC infection showed higher nitrite levels compared to LII one (S1B Fig).

Analysis of ARG in spleens from both mouse lineages revealed an overall decrease in the enzyme activity compared to noninfected controls. For BALB/c mice infected by the LIC strain at 30 dpi, there was a significant decrease (p < 0.0001) in ARG activity in comparison with the corresponding noninfected control (Fig 1D), while such a decrease was not observed in those animals infected by the LII strain. In fact, mice infected by the LII strain showed higher ARG activity (p < 0.0001) than those infected by the LIC strain. However, at 60 dpi, a decrease in ARG activity (p ≤ 0.0001) was observed in BALB/c mice infected with LIC or LII strains when compared with noninfected mice. Nevertheless, mice infected by the LII strain still showed more ARG activity (p ≤ 0.05) than those infected by the LIC strain. In Swiss Webster mice, the ARG activity was significantly smaller in groups infected by the LIC and LII strains than in the uninfected control at both 30 and 60 dpi. In addition, no differences were observed between the two infected groups. Interestingly, in assays with liver cell cultures, a significant decrease (p ≤ 0.0001) in ARG was observed in both mouse lineages infected by LIC or LII strains at 30 and 60 dpi (S1C Fig).

### *In vitro* biological behaviour of LII and LIC

**Fig 2:**
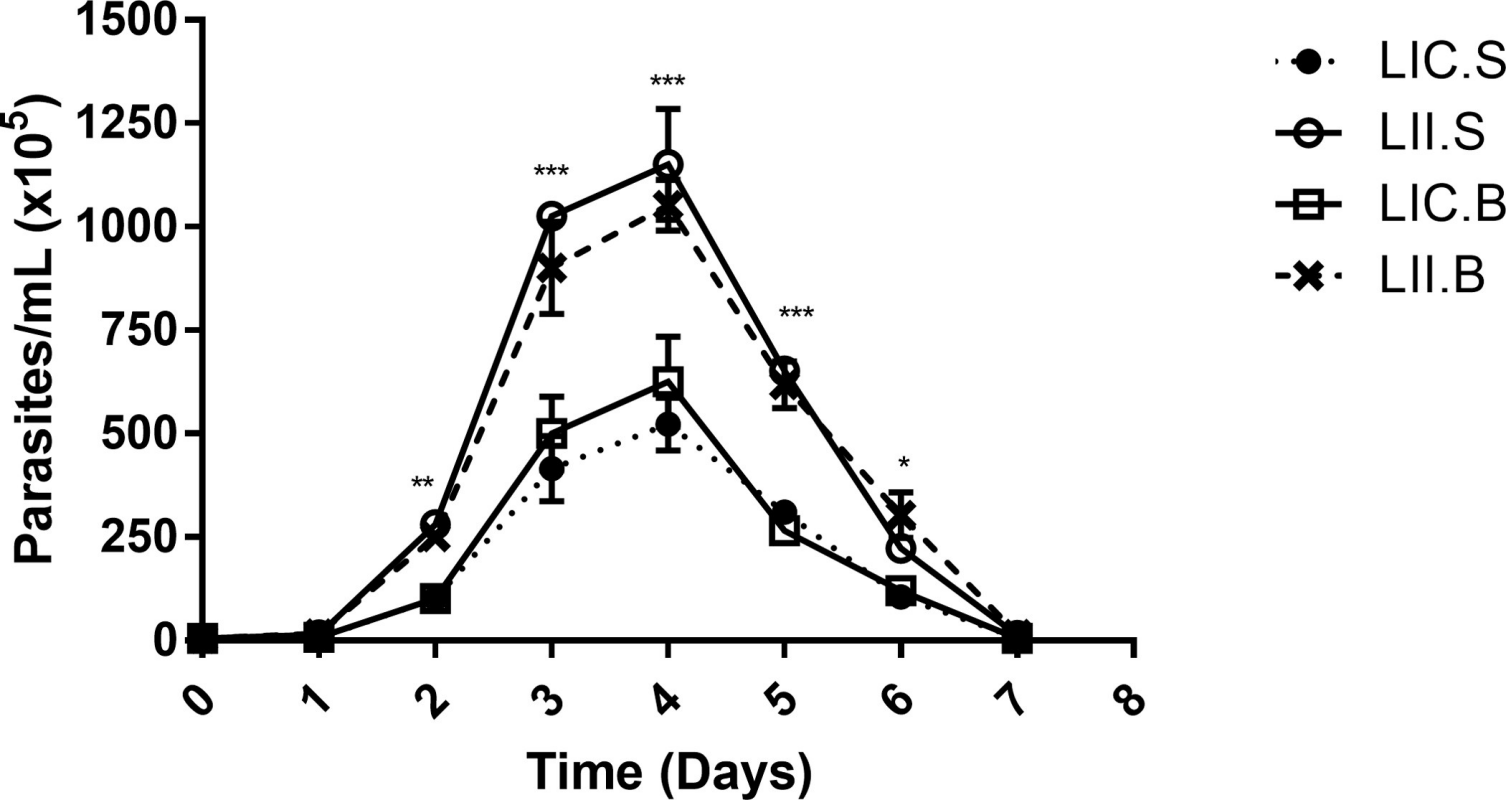
*In vitro* proliferation of promastigote forms. Promastigotes were obtained from amastigotes freshly isolated from the four infected experimental groups and cultivated for 7 days. The parasites were counted daily, and the growth profile was evaluated. LIC.B (*L*. *infantum chagasi* isolated from BALB/c mice); LII.B (*L*. *infantum infantum* isolated from BALB/c mice); LIC.S *(L*. *infantum chagasi* isolated from Swiss Webster mice); and LII.S *(L*. *infantum infantum* isolated from Swiss Webster). *p ≤ 0.05; **p ≤ 0.009; ***p < 0.0001. The values are represented by mean ± standard deviation of 3 independent experiments realized in experimental triplicate.

Since *L*. *infantum* strains (*L*. *infantum infantum* and *L*. *infantum chagasi*) showed differences in infectivity in the two murine lineages evaluated, our next step was to analyze their *in vitro* behavior and the potential influence of the host on these strains through promastigotes proliferation and metacyclogenesis. First, promastigotes were obtained from amastigotes from the four infected groups generating the isolates: LIC.B (*L*. *infantum chagasi* isolated from BALB/c mice), LII.B (*L*. *infantum infantum* isolated from BALB/c mice), LIC.S *(L*. *infantum chagasi* isolated from Swiss Webster mice), and LII.S *(L*. *infantum infantum* isolated from Swiss Webster mice). In general, the proliferation profiles of the two *Leishmania* strains were similar, since the late log phase was between the 3^rd^ and 4^th^ days of growth. However, the parasite concentration of LII (LII.B and LII.S) was higher than that of LIC (LIC.B and LIC.S), with significant differences from the 2^nd^ to 6^th^ days of culture (Fig 2).

Although the four isolates presented the same growth profile, their proliferation rates were different. Therefore, the next step was to evaluate whether these parasites present differences in percentage of metacyclic forms in culture, using the complement lysis test at different times (48, 72 and 96 h). At all times evaluated, the percentage of complement-resistant cells was significantly higher in *L*. *infantum infantum* isolated from BALB/c and Swiss Webster mice, suggesting that *L*. *infantum infantum* could be more infective than *L*. *infantum chagasi*, independent of the mouse lineage from which it was isolated (Table 1). It is important to note that the highest percentage of metacyclic forms was seen at 72 h, coinciding with the late log phase of the growth curve.

**Table 1:** Evaluation of the metacyclogenesis for the four *Leishmania* isolates.

% metacyclic parasites				
Time	BALB/c		Swiss Webster	
	LII.B	LIC.B	LII.S	LIC.S
**48h**	56.0 ± 5.7**	27.0 ± 3.0	49.0 ± 7.8*	22.0 ± 3.5
**72h**	75.0 ± 3.5**	38.0 ± 2.0	68.0 ± 4.2**	34.0 ± 6.4
**96h**	56.0 ± 1.4*	31.0 ± 1.4	43.0 ± 6.4*	18.0 ± 1.4

Percentage of metacyclic forms was determined by complement lysis test. LIC.B (*L*. *infantum chagasi* isolated from BALB/c mice); LII.B (*L*. *infantum infantum* isolated from BALB/c mice); LIC.S *(L*. *infantum chagasi* isolated from Swiss Webster mice); LII.S *(L*. *infantum infantum* isolated from Swiss Webster mice).

*p ≤ 0.0009;

**p < 0.0001. Comparison of metacyclic form percentages observed on LII and LIC isolates from each mouse lineage. The values are represented by mean ± standard deviation of 3 independent experiments realized in experimental triplicate.

**Fig 3:**
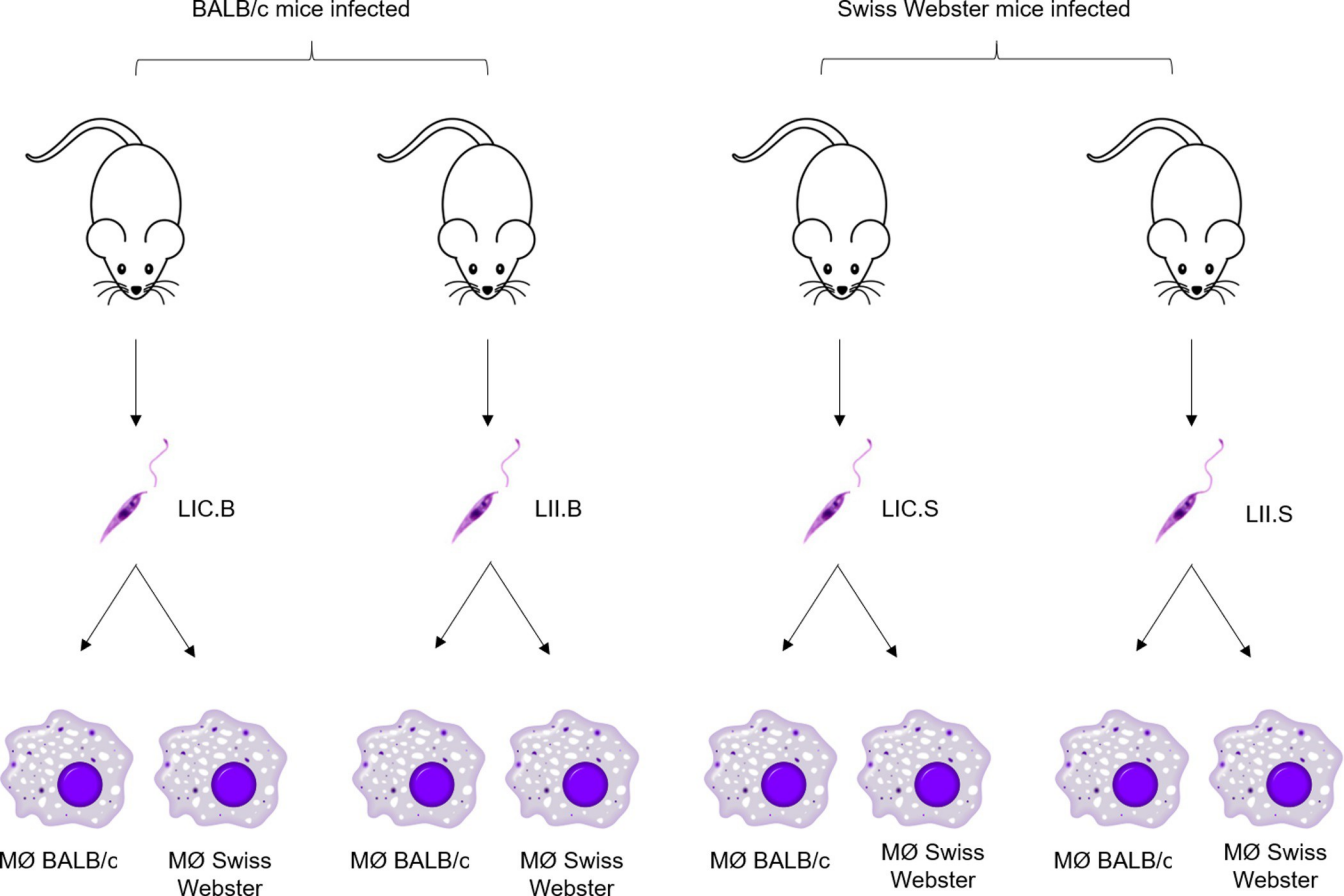
Workflow of *in vitro* infection. Four *L*. *infantum* isolates were used to infect peritoneal macrophages from BALB/c and Swiss Webster mice, at a ratio of 5:1 (parasite/macrophage). The infections were maintained for 24 or 72 h. MØ (macrophage); LIC.B (*L*. *infantum chagasi* isolated from BALB/c mice); LII.B (*L*. *infantum infantum* isolated from BALB/c mice); LIC.S *(L*. *infantum chagasi* isolated from Swiss Webster mice); and LII.S *(L*. *infantum infantum* isolated from Swiss Webster mice).

To confirm the hypothesis that the LII strain is more infective than the LIC strain, the four parasite isolates from BALB/c and Swiss mice were used to infect peritoneal macrophages obtained from the same mice from which they were isolated (Fig 3). After 24 or 72 h, the infection index was calculated.

**Fig 4:**
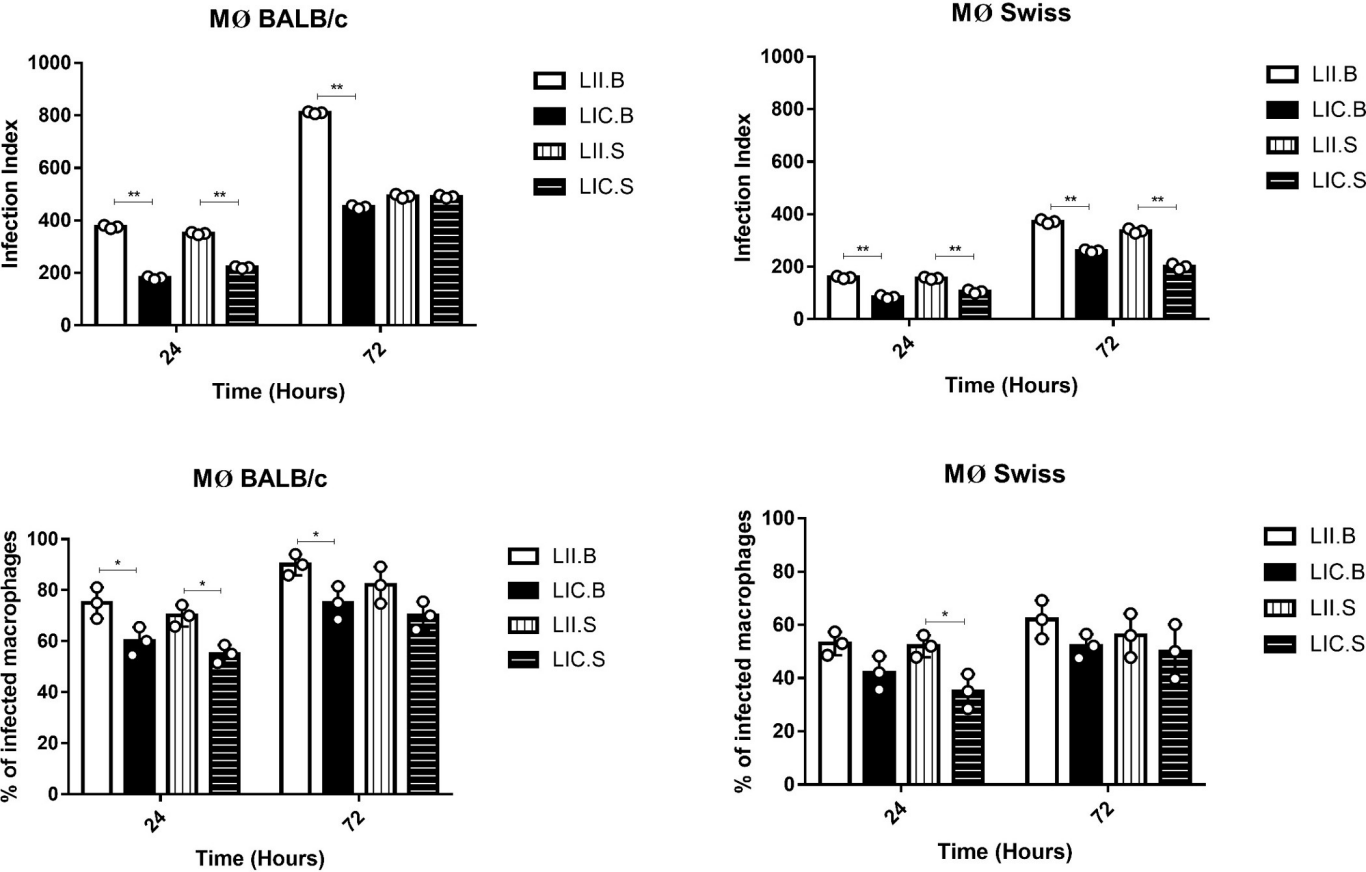
Infectivity of the four *Leishmania* isolates to peritoneal macrophages. (A) Infection index in macrophages from BALB/c mice (top) and percentage of infected macrophages (bottom). (B) Infection index in macrophages from Swiss Webster mice (top) and percentage of infected macrophages (bottom). Peritoneal murine macrophages were infected (overnight) by stationary-phase promastigotes (MOI: 5:1), and cultures were maintained for 24 or 72 h. On each coverslip, at least 200 cells were randomly counted, and infection index was calculated using the following formula: % infected macrophages x number of amastigotes)/total number of macrophages. MØ (macrophage); LIC.B (*L*. *infantum chagasi* isolated from BALB/c mice); LII.B (*L*. *infantum infantum* isolated from BALB/c mice); LIC.S *(L*. *infantum chagasi* isolated from Swiss Webster mice); and LII.S *(L*. *infantum infantum* isolated from Swiss Webster mice). *p ≤ 0.05; *p < 0.0001. The values were represented by mean ± standard deviation of 3 independent experiments realized in experimental triplicate.

As expected, infection rates were higher at 72 h than at 24 h (Fig 4A and 4B). In macrophages from BALB/c mice (Fig 4A), the LII strain from both mouse lineages (LII.B and LII.S) was more infective than the LIC strain at both 24 and 72 h and presented the highest percentages of infected macrophages (except LII.S at 72h). In macrophages from Swiss Webster mice, the LII strain again proved to be more infective than the LIC strain at both times analyzed, but only the LII.S at 24 h showed a significant difference in the percentage of infected (Fig 4B). Comparing the mouse lineages, macrophages from BALB/c mice were more susceptible to infection than those from Swiss Webster mice based on the higher infection rates. These data reinforced those obtained in metacyclogenesis and *in vivo* experiments.

### NOS activity

**Fig 5:**
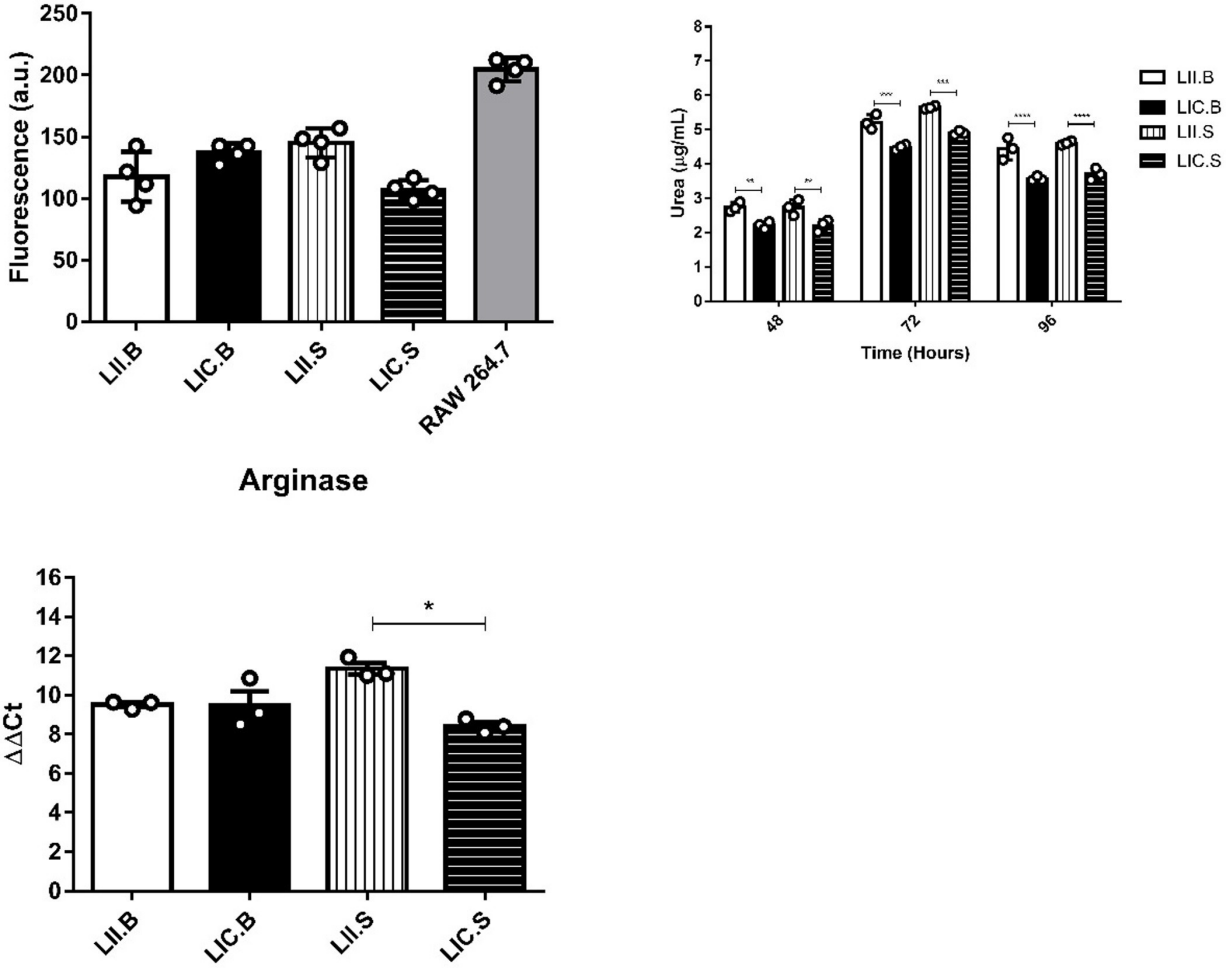
*In vitro* NOS and ARG activities. (A) NOS activity of promastigotes. (B) ARG activity by urea production by promastigotes. (C) Relative expression of the arginase gene. The intracellular NO production was detected by DAF-2DA labeling. RAW 264.7 macrophages were used as the positive control. The urea production was measured by spectrometry at 540 nm. The relative expression of the arginase gene was evaluated by qRT-PCR. The alfa-tubulin gene was used as the reference gene. LIC.B (*L*. *infantum chagasi* isolated from BALB/c mice); LII.B (*L*. *infantum infantum* isolated from BALB/c mice); LIC.S *(L*. *infantum chagasi* isolated from Swiss Webster mice); and LII.S *(L*. *infantum infantum* isolated from Swiss Webster mice). *p ≤ 0.05; **p ≤ 0.009; ***p ≤ 0.0009; ****p < 0.0001. The values are represented by mean ± standard deviation of 3 independent experiments realized in experimental triplicate.

NOS activity was evaluated in promastigotes derived from amastigotes isolated from the four experimental groups, i.e., LII.B, LIC.B, LII.S and LIC.S. Interestingly, the intracellular NO production in the four isolates showed no significant differences, possibly because the LII and LIC strains produced similar basal NO levels (Fig 5A).

### ARG activity and gene expression

ARG activity in promastigotes of the four isolates was measured after 48 to 96 h in culture. The highest enzyme levels were obtained at 72 h of culture. At all evaluated points, LII isolated from BALB/c and Swiss Webster mice (LII.B and LII.S) showed higher urea levels compared to LIC, demonstrating the highest ARG activity (Fig 5B). Higher enzyme levels occurred in LII parasites with higher percentages of metacyclic forms, suggesting that ARG activity may be related to the parasite infectivity. Since LII and LIC strains showed differences in the *in vitro* ARG activity, the expression of this enzyme was evaluated in the four *Leishmania* isolates. As observed, LII.S parasites (*L*. *infantum infantum* isolated from Swiss Webster) showed an increase (p ≤ 0.05) in ARG expression compared to LIC.S (*L*. *infantum chagasi* isolated from Swiss Webster) (Fig 5C), indicating that such activity differences may be associated with the enzyme gene. Thus, to identify genetic differences in the ARG gene, Sanger sequencing was performed. However, upon analyzing the four *Leishmania* isolates, no differences were observed between the ARG sequences of the LII and LIC strains. Thus, it is possible to state that the strains are molecularly equal (relative to the ARG gene) and identical to the *L*. *infantum* sequence deposited in the TriTrypDB database.

## Discussion and conclusions

Visceral leishmaniasis (VL) is a tropical disease that affects millions of people and can lead to death. Therefore, the study of this pathology, as well as the understanding of the characteristics of its etiological agents, are of fundamental importance. As previously reported, the classification of the parasite responsible for American visceral leishmaniasis (AVL) is still controversial. Considering *L*. *infantum chagasi* (LIC) as a different species or as a synonymous of *L*. *infantum infantum* (LII) must take into account the biological, biochemical and pathogenic behaviors [29]. In the literature, there are several studies demonstrating specific genetic and molecular variations among *L*. *infantum* isolates from different geographic regions [30]. However, few studies have addressed the biological differences and the behavior of these strains during infection. In this work, it was reported that LIC and LII strains presented differences in infectivity and survival capacity in two different murine models.

Genetic factors dependent on the mouse lineage interfere with the success of experimental infection, leading to differences in disease development. Thus, the choice of animal model is of great relevance since the genetic background influences both immunological and pharmacological responses to chemotherapeutic agents. It has already been established that different elements of the host’s immune response directly influence the course of infection [31, 32]. In the *L*. *major* infection model in C57BL/6 and BALB/c mice, the T-helper cell-mediated response plays a key role in infection control [33]. Such an immune response is determined by environmental and genetic factors, explaining different susceptibilities to infection in mice with different genetic backgrounds. In the present study, BALB/c mice (inbred lineage) were more susceptible to *Leishmania* infantum infection than Swiss Webster mice (outbred lineage).

Parasites of the genus *Leishmania* have the ability to modulate the infected host’s immune response to increase their chances of intracellular survival [34]. One of these mechanisms is the uptake of L-arginine, a substrate used by the enzyme iNOS, in order to suppress NO production in the host and to inhibit the release of pro-inflammatory cytokines [35]. This process leads to an increase in ARG activity, which in turn can be modulated by the parasite. The data here obtained demonstrated that spleen and liver cells from infected mice produced different NO levels, with the highest levels observed in LIC strain-infected BALB/c cells. Interestingly, although infected spleen cells produced more NO than uninfected ones, both the LIC and LII strains were still able to survive and multiply within the host, increasing parasite load at 60 dpi, especially in the case of the LII strain. Marques and collaborators (2015) [36] have reported that LII parasites were able to survive high amounts of NO added to the culture. Regarding ARG activity, lower urea production was observed in the spleen and liver of infected animals when compared to uninfected controls. However, spleen cells from BALB/c infected by the LII strain showed higher urea production than the other infected groups. This higher ARG production may be correlated with higher parasite loads and parasite survival observed in spleen cells. Taken together, the results of NOS and ARG activities suggest that different strains of *L*. *infantum* may influence the infected host L-arginine metabolism in different ways.

In addition to the host's ability to respond to infection, the biological differences between the parasite strains and parasite species, should be considered. Previous studies have shown that *L*. *infantum* isolates belonging to the same zymodeme may present different sensitivities to the host's immune response. This may be related to parasite virulence factors [29]. In addition, there are studies indicating that differences in infectivity between *L*. *infantum* strains could be related to parasite modulation of the L-arginine metabolic enzyme expression during mice infection [34]. Studies evaluating differences between parasites from different geographic regions are needed. Therefore, the present study aimed to evaluate some biological and molecular characteristics of different *L*. *infantum* strains and their behavior when isolated from different hosts (LIC.B, LII.B, LIC.S and LII.S). Both LII isolates (LII.B and LII.S) showed a higher growth rate than LIC.B and LIC.S, even considering that all four isolates present the same proliferation profile. The infectivity and behavior of these parasites during infection in macrophages from the same hosts from which they were isolated were also evaluated. As in the *in vivo* results, the LII strain was shown to be more infective by the percentage of metacyclic forms in culture, as well as by the infection index in murine macrophages. In addition, macrophages from mice with different genetic backgrounds also presented different susceptibilities to *in vitro* infection.

As reported, *Leishmania* can modulate the host’s L-arginine metabolism. Therefore, it is of great interest to establish a relationship between the levels of NOS and ARG induction and inhibition in intracellular parasites, as well as in the infected host cell itself. However, few studies have reported differences in the L-arginine metabolic enzymes activity, mainly comparing promastigotes from different strains of *L*. *infantum*. Thereby, the intracellular NO production by promastigotes of *L*. *infantum* strain isolates from different hosts was evaluated using the fluorescent indicator DAF-2DA. However, no significant differences were observed among the four isolates. As demonstrated by Balestieri and collaborators (2002) [37], inhibition of iNOS activity was observed in *Leishmania*-infected macrophages followed by reduction in NO production, indicating the parasite’s ability to evade host cell defense mechanisms.

The existence of NOS in *Leishmania* spp. [6], as well as in *Trypanosoma cruzi* [38], has already been reported. However, the identification of the gene responsible for encoding this enzyme in trypanosomatids [39] is insufficient in the literature and in genomic databases, making it difficult to search for molecular differences in NOS among *Leishmania* strains. However, the ARG gene is already well described, thus allowing several analyses to be performed. In this work, it was demonstrated that *L*. *infantum* strains isolated from different hosts presented distinct ARG activities during their proliferation. However, LII and LIC strains showed higher enzyme activity on the third day of culture, when parasites reached the stationary phase. To search for possible differences in ARG gene sequences and in relative enzyme expression, Sanger sequencing and qPCR assays were performed. As a result, it was found that the ARG gene sequence was identical in LIC and LII strains and identical to the sequence deposited in the online database TriTryp. However, the strains isolated from different hosts presented different mRNA expression levels relative to each other, which may be related to posttranscriptional changes.

Data presented in this study indicate that strains of *L*. *infantum* from the Old and New Worlds present different biological behaviors, although they are considered identical by some authors. This demonstrates the importance of conducting further studies on these differences, as this may assist in the search for better alternatives to combat these parasites.

It was also concluded that mice with different genetic backgrounds present different susceptibilities to infection by *L*. *infantum* strains, both *in vivo* and *in vitro*. This reinforces the importance of the search for ideal animal models to study the leishmaniasis pathophysiology. Before starting a study, it is necessary to verify how each animal model behaves during infection by a certain pathogen. This leads to a better control of the host’s interference in the experimental phenomenon to be analyzed, when this interference is not part of the objectives. In addition, *L*. *infantum* strains demonstrated differences in L-arginine metabolism, which should be better studied during the host-parasite relationship. These differences may be related to disease pathogenesis since the nitric oxide synthase and arginase enzymes have been related to the modulation of the host's response to infection.

## Supporting information

S1 Fig Evaluation of *in vivo* infection of two *L*. *infantum* strains. (A) Parasite load in the livers of infected mice. (B) Nitrite levels (NOS activity) of liver cell cultures. (C) Urea levels (ARG activity) of liver cell cultures. BALB/c and Swiss Webster mice infections were maintained at 30 or 60 dpi. The number of parasites/mg of tissue was estimated based on total weight of the liver removed and the parasite load in the serial dilution. The nitrite and urea levels were measured by spectrophotometry at 540 nm. Control: noninfected mice; LII (*L*. *infantum infantum*); LIC (*L*. *infantum chagasi*). *p ≤ 0.05; **p ≤ 0.009; ***p < 0.0001. The values are represented by mean ± standard deviation of 3 independent experiments with 5 animals in each group.
(TIF)
