# Supplementary figures and images for: Comparative analysis of biological aspects of Leishmania infantum strains
Source: PLoS One. 2020 Dec 3;15(12):e0230545. doi: 10.1371/journal.pone.0230545 (PMC7714135; doi:10.1371/journal.pone.0230545)

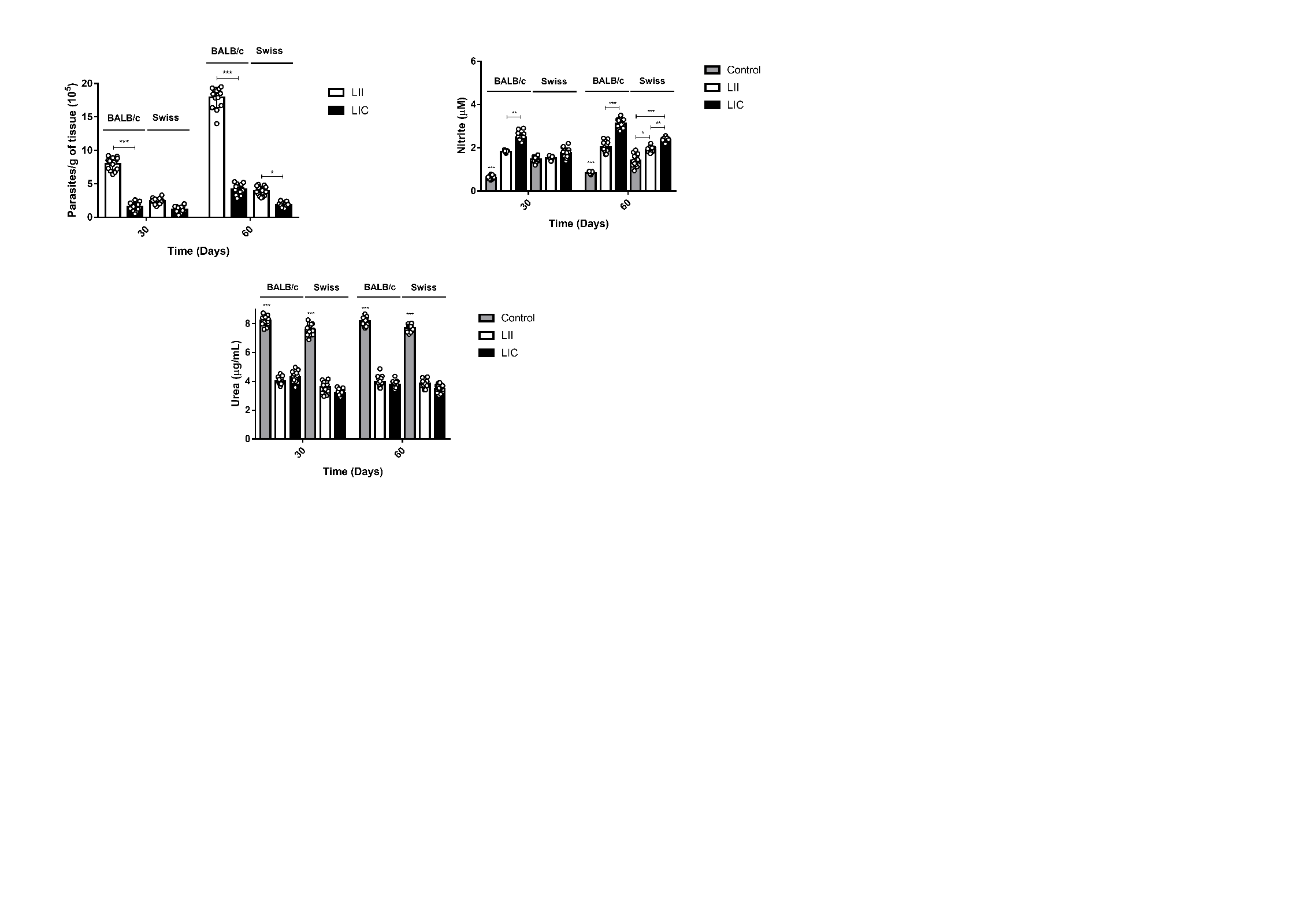

Supplement: S1 Fig — (A) Parasite load in the livers of infected mice. (B) Nitrite levels (NOS activity) of liver cell cultures. (C) Urea levels (ARG activity) of liver cell cultures. BALB/c and Swiss Webster mice infections were maintained at 30 or 60 dpi. The number of parasites/mg of tissue was estimated based on total weight of the liver removed and the parasite load in the serial dilution. The nitrite and urea levels were measured by spectrophotometry at 540 nm. Control: noninfected mice; LII (L. infantum infantum); LIC (L. infantum chagasi). *p ≤ 0.05; **p ≤ 0.009; ***p < 0.0001. The values are represented by mean ± standard deviation of 3 independent experiments with 5 animals in each group. (TIF) [file pone.0230545.s001.tif]
